# *In vitro* rumen biohydrogenation of unsaturated fatty acids in tropical grass-legume rations

**DOI:** 10.14202/vetworld.2020.661-668

**Published:** 2020-04-12

**Authors:** Malik Makmur, Mardiati Zain, Fauzia Agustin, Riesi Sriagtula, Ezi Masdia Putri

**Affiliations:** Department of Animal Nutrition, Faculty of Animal Science, Andalas University, Kampus Limau Manis, Padang, West Sumatera, Indonesia

**Keywords:** biohydrogenation, *in vitro* rumen, tropical grass-legume rations, unsaturated fatty acid

## Abstract

**Aim:**

The aim of this study was to evaluate the effects of various combinations of tropical grass-legume species in rations on the biohydrogenation (BH) activity of unsaturated fatty acids (FAs), C18:0 composition, and fermentation profile in an *in vitro* rumen system.

**Materials and Methods:**

Samples of the following five fodder plants were used: One species of grass (*Pennisetum purpureum*) and four species of tree legumes (*Leucaena leucocephala*, *Gliricidia sepium*, *Calliandra calothyrsus*, and *Indigofera zollingeriana*). The following eight experimental diets were evaluated: 50% *P. purpureum* + 50% *L. leucocephala* (LL I); 50% *P. purpureum* + 50% *G. sepium* (GS I); 50% *P. purpureum* + 50% *C. calothyrsus* (CC I); 50% *P. purpureum* + 50% *I. zollingeriana* (IZ I); 75% *P. purpureum* + 25% *L. leucocephala* (LL II); 75% *P. purpureum* + 25% *G. sepium* (GS II); 75% *P. purpureum* + 25% *C. calothyrsus* (CC II); and 75% *P. purpureum* + 25% *I. zollingeriana* (IZ II). Each ration was replicated 3 times. *In vitro* rumen incubation was performed for 48 h, according to the Tilley and Terry method. Determination of the FA profiles of the forage materials and rumen fluid samples was performed using gas chromatography.

**Results:**

The percentage of polyunsaturated FA (PUFA) in the forage materials ranged from 34.18% (*P. purpureum*) to 74.51% (*C. calothyrsus*). The percentage of monounsaturated FA (MUFA) ranged from 5.06% (*P. purpureum*) to 8.71% (*L. leucocephala*). The percentage of saturated FA (SFA) was the lowest at 19.12% (*C. calothyrsus*) and highest at 60.76% (*P. purpureum*). *In vitro* BH of C18:3 n-3, C18:2 n-6, C18:1 n-9, and C18 PUFA in the experimental diets ranged from 72% to 100%. The BH of C18:1 n-9 in GS I (80%) and IZ I (72%) was significantly different (p<0.05). The percentage of C18:0 was 10-50% and significantly different (p<0.05) among treatments, with the highest (of 50%) in GS II. No significant differences (p>0.05) were observed in the fermentation parameters (pH, total volatile FAs, *in vitro* dry matter digestibility, and *in vitro* organic matter digestibility) among the treatments, except in NH_3_ concentration (p<0.05).

**Conclusion:**

The various combinations of tropical legumes do not have significant inhibitory effects on the BH of C18:2 n-6, C18:3 n-3, and C18 PUFA after *in vitro* incubation for 48h. Furthermore, an increase in the tropical legume ratio in the ration tends to suppress C18:0 formation after the fermentation simulation process. IZ I has the potential to reduce C18:1 n-9 (MUFA) disappearance and yield an ideal rumen fermentation profile.

## Introduction

Currently, the main challenge in ruminant nutrition research is to derive food products from ruminants with fatty acid (FA) profiles that have acceptable health standards. Unsaturated FA (UFA) groups, such as polyunsaturated FAs (PUFAs) and monounsaturated FAs (MUFAs), for example, linolenic acid (C18:3 n-3), linoleic acid (C18:2 n-6), oleic acid (C18:1 n-9), and conjugated linoleic acids (*cis*-9 *trans*-11 C18:2,), have medical effects and are main targets for the effective deposition of FAs in meat and dairy products [1,2]. In ruminants, FA metabolism in the rumen is initiated by lipolysis (which liberates the lipid fraction in the feed into free FA), followed by biohydrogenation (BH), performed by two dominant microbial groups, namely, *Butyrivibrio fibrisolvens* and *B. proteoclasticus*, to transform most UFA into saturated FAs (SFAs), especially stearic acid (C18:0), which promotes degenerative diseases in humans [3].

The manipulation of rumen BH using a forage-based ration approach elucidates two important points: (1) UFA concentrations in tropical forage species and (2) presence of BH reduction agents in the form of plant secondary metabolites. Forage is the main source of FA intake for ruminants because it has a high UFA composition (>50% FA) in chloroplast lipids, thus, has a beneficial FA profile at the level of body tissue and lipid distribution in ruminants, in addition to the reduction of volatile components and minimization of off-flavor in meat [4-6]. In addition, the presence of plant secondary metabolites, such as phenol, tannin, flavonoid, and saponin components, suppresses the activity of rumen BH at various stages of isomerization and saturation through the modulation of the rumen microbiota [7-9]. This increases the preservation of UFA and accumulation of BH intermediate metabolites in the rumen digesta.

The previous *in vitro* and *in vivo* studies have indicated that the phenol and tannin contents in forage are natural indicators of the potential of a feed crop as a BH reduction agent and the anti-methanogenic activity of tropical legumes [10-12]. Thus, tropical legume compositions in the ration are important for sustainable, inexpensive, and adaptable strategies for the protection of UFA from rumen degradation. However, there is limited information on the effects of tropical grass-legume levels on the ration and on the BH of UFA.

The aim of this study was to evaluate the effects of various combinations of tropical grass-legume species in rations on UFA BH activity, C18:0 composition, and fermentation profile in an *in vitro* system.

## Materials and Methods

### Ethical approval

In this experiment, we did not use live animals; thus, ethical approval is not required.

### Study area, forage materials, and nutrition analysis

Plant species samples were collected from the pasture region (±25 ha) of UPT Teaching Farm, Faculty of Animal Science, Andalas University, Padang, Indonesia. The geographical position of the area is 0°54’30”S and 100°27’48”E at an elevation of 250m above sea level. The average rainfall is >4000mm/year and the climate type is wet. The soil profile in the area around the pasture is Ultisol type. Samples of five fodder plants were collected: One species of grass (*Pennisetum purpureum*) and four species of tree legumes (*Leucaena leucocephala*, *Gliricidia sepium*, *Calliandra calothyrsus*, and *Indigofera zollingeriana*) that had entered the generative phase. These plant species are used extensively in forage rations for ruminants by farmers in Indonesia. For tree legumes, leaves, flowers, and soft stems (edible parts) were sampled and as much as 3kg of fresh material was used. After collection, the samples were maintained indoors at 28°C for 24h, oven-dried at 60°C for 3h, and milled to pass through a 1mm sieve. Then, the samples were placed in an airtight plastic bag and stored in a refrigerator at 4°C.

The determination of the nutritive value of forage samples (Table-1) (crude protein, ether extract, and crude fiber contents) was carried out using proximate analysis [13]. The analysis of neutral detergent fiber, acid detergent fiber, and lignin contents followed the protocols of Goering and Van Soest [14]. The measurement of the gross energy of plant samples was performed using a bomb calorimeter [15]. The quantification of plant secondary metabolites (total phenols, tannins, and saponins) was performed according to the procedure of Makkar [16] and Hiai *et al*. [17] using a UV-visible U-1800-5930482 spectrophotometer (High-Technologies Corporation).

**Table-1:** Nutrition content of forage materials.

Nutrition content	Forage materials					SEM

	*P. purpureum*	*L. leucocephala*	*G. sepium*	*C. calothyrsus*	*I. zollingeriana*	
Crude protein^A^	7.02	25.15	25.20	28.46	31.90	4.31
Ether extract^A^	2.51	4.80	3.96	4.11	3.64	0.37
Energy^B^	37.63	42.58	40.90	42.81	40.76	0.93
Crude fiber^A^	34.12	15.24	13.09	11.50	9.95	4.42
Ash^A^	8.18	8.56	9.07	7.04	9.43	0.41
NDF^A^	64.01	31.63	35.73	50.72	21.91	7.43
ADF^A^	34.19	23.39	22.47	31.45	8.37	4.51
Lignin^A^	3.48	9.14	6.68	12.96	1.62	2.02
Cellulose^A^	28.36	14.85	15.24	16.98	6.09	3.56
Total phenolic^A^	1.98	2.65	1.11	0.66	2.46	0.38
Tannin^A^	0.94	1.15	0.19	0.16	1.13	0.22
Saponin^A^	0.00	2.2	0.00	1.78	2.5	0.54

A g/100 g dry matter.

B kcal/kg. SEM=Standard error of the mean

### Animals and *in vitro* method

Rumen liquor from Kacang crossbred goats (body weight, ±20kg) was used as the fermentation medium. The goats were obtained from a slaughterhouse in Padang City. Before slaughter, the goats were fed roughage *ad libitum*. The incubation procedure was performed *in vitro* for 48h according to the Tilley and Terry method [18]. The following combinations of the grass-legume species were used for *invitro* experimental ration I: 50% *P. purpureum* + 50% *L. leucocephala* (LL I); 50% *P. purpureum* + 50% *G. sepium* (GS I); 50% *P. purpureum* + 50% *C. calothyrsus* (CC I); and 50% *P. purpureum* + 50% *I. zollingeriana* (IZ I). The following combinations were used for *in vitro* experimental ration II: 75% *P. purpureum* + 25% *L. leucocephala* (LL II); 75% *P. purpureum* + 25% *G. sepium* (GS II); 75% *P. purpureum* + 25% *C. calothyrsus* (CC II); and 75% *P. purpureum* + 25% *I. zollingeriana* (IZ II). Each treatment was replicated 3times.

The main reason for selecting *P. purpureum* is that the species has a higher content of BH reduction agents (total phenols and tannins) than other tropical grass species [6]. Next, 2.5g of the experimental diet was weighed and placed in a 250ml fermenter tube. Then, 50ml of rumen fluid and 200ml of McDougall buffer solution (9.8g of NaHCO_3_, 9.3g of Na_2_HPO_4_.12 H_2_O, 0.57g of KCl, 0.47g of NaCl, 0.06g of MgSO_4_.7H_2_O, and 0.04g of CaCl_2_.2H_2_O per liter of distilled water) were poured into the fermenter tube (1:4 ratio), CO_2_ gas was injected into the tube for 30 s, and the tube was sealed with a rubber cap. The blank was prepared by adding only rumen fluid to three fermenter tubes. To obtain the FA profile at 0h, we used three 50ml plastic tubes filled with rumen stored at 0°C. Each fermenter tube was placed in a shaker bath at 39°C and constantly rotated at 100rpm for 48h. Then, the fermenter tubes were immersed in ice water to stop the fermentation process. The rubber cap was removed to measure the pH of the rumen fluid with a pH meter and the fermented liquid was centrifuged at 1000 g for 5min. The supernatant was collected for FA determination, total volatile FAs (VFAs) was measured using the distillation method [19], and NH_3_ production was measured using the microdiffusion method [20]. The sediment was filtered using Whatman paper no.41 and dried in an oven at 60°C for 24h to calculate the digestibility of dry matter and organic feed *in vitro* according to the procedures of the Association of Official Analytical Chemists (AOAC) [21].

### FA determination

FA methyl ester (FAME) standard solution was used (Sigma-Aldrich, Inc., USA) to determine the composition of plant FA in rumen fluid. Then, the fat content in the samples was determined as follows: (1) Direct extraction with the Soxhlet apparatus for solid samples and (2) extraction from liquid samples using the hydrolysis method. The fat extract was prepared for the methylation process (cold extraction). The procedures for making standard solutions, sample preparation (determination of fat content), and extraction of sample fat for methylation were in accordance with the procedures for FAME by the American Oil Chemists’ Society [22] and AOAC [21]. FAME was injected into the gas chromatography (GC) instrument at an injector temperature of 225°C. The quantification of the FA component in the sample was performed using GC Agilent 7890B (Agilent Technologies, Inc., USA) equipped with a capillary column of 100 m×0.25 mm×0.2 μm (Supelco SPTM 2560). FAs were detected in the column using a flame ionization detector at 240°C. The carrier gas used was N_2,_ with a flow rate of 18.0cm/s at a 1:100 split ratio. The fraction and form of FA (*cis*, *trans*, SFA, MUFA, and PUFA) were determined by adjusting the retention time of methyl FA to the FAME standard, according to the protocol of Ratnayake *et al*. [23]. The FA concentration is expressed as the percentage of total FA.

### Rumen BH calculations

The *in vitro* rumen BH activity was calculated by measuring the differences between FA components at the end of incubation (48h) and before incubation (0h), according to the methods of Jayanegara *et al*. [11] and Jafari *et al*. [24]:

BH C18:3 n-3 (%) = [(C18:3 n-3_0h_ – C18:3 n-3_48h_)/C18:3 n-3_0h_] × 100

BH C18:2 n-6 (%) = [(C18:2 n-6_0h_ – C18:2 n-6_48h_)/C18:2 n-6_0h_] × 100

BH C18:1 n-9 (%) = [(C18:1 n-9_0h_ – C18:1 n-9_48h_)/C18:1 n-9_0h_] × 100

BH PUFA (%) = [(PUFA_0h_ – PUFA_48h_)/PUFA_0h_] × 100

Specifically, the composition of C18:0(48h), the main end-product of the overall metabolism of UFA (lipolysis and BH) in the rumen, was determined using the following formula:

C18:0 (%) = [C18:0_48h_/total C18 FA_48h_] × 100

In this study, we also aimed to detect intermediate BH FAs, namely, *trans*-C18:2 n-6; however, this intermediate metabolite was not detected after *in vitro* incubation for 48h (limit of detection, 0.00164%).

### Statistical analysis

The study design was completely randomized. The BH activity data and fermentation parameters were evaluated in JASP version0.9.2 (University of Amsterdam, Amsterdam, Netherlands) [25]. If the treatments were significantly different (p<0.05), Fisher’s least significant difference test was performed.

## Results

### FA profiles in the forage material

The total concentration of FA (% ether extract) in the tropical forage ranged from 2.37% (*P. purpureum*) to 4.45% (*G. sepium*), with an average of 3.90% (Table-2). The percentage of PUFA in the forage material ranged from 34.18% (*P. purpureum*) to 74.51% (*C. calothyrsus*). Ahigh percentage of PUFA was also observed in *I. zollingeriana*, *G. sepium*, and *L. leucocephala*. The percentage of MUFA ranged from 5.06% (*P. purpureum*) to 8.71% (*L. leucocephala*). In addition, the percentage of SFA was the lowest at 19.12% (*C. calothyrsus*) and highest at 60.76% (*P. purpureum*). The highest proportion of C18:3 n-3 (>70% total FA) was observed in *C. calothyrsus*, followed by *I. zollingeriana*. The lowest proportion of C18:3 n-3 was observed in *L. leucocephala* and *P. purpureum*. The highest proportion of C18:2 n-6 was detected in *C. calothyrsus* and *L. leucocephala* and the lowest in *G. sepium* and *P. purpureum*. Furthermore, the highest proportion of C18:0 was observed in *G. sepium* and *L. leucocephala* and the lowest in *C. calothyrsus*.

**Table-2:** Fatty acid composition of forage materials for *in vitro* fermentation.

Fatty acids	Forage materials					SEM

	*P. purpureum*	*L. leucocephala*	*G. sepium*	*C. calothyrsus*	*I. zollingeriana*	
C14:0^A^	1.69	2.35	1.12	0.25	1.37	0.01
C16:0^A^	22.78	19.53	21.12	6.37	17.16	0.12
C18:0^A^	4.64	8.00	8.76	2.70	4.12	0.06
C18:1n-9^A^	5.06	8.47	4.94	4.66	4.35	0.04
C18:2n-6^A^	12.66	13.65	11.69	20.83	15.33	0.09
C18:3n-3^A^	20.25	36.24	43.15	53.68	47.83	0.31
C20:0^A^	6.75	2.35	2.47	1.96	1.83	0.01
SFA^A^	60.76	41.41	39.33	19.12	32.27	0.18
MUFA^A^	5.06	8.71	5.17	6.37	4.35	0.04
PUFA^A^	34.18	49.88	55.73	74.51	63.16	0.39
Total FA^B^	2.37	4.25	4.45	4.08	4.37	0.39

A % total FA.

B % ether extract. SEM=Standard error of the mean, *P. purpureum=Pennisetum purpureum,*

L. leucocephala=Leucaena leucocephala, G. sepium=Gliricidia sepium, C. calothyrsus=Calliandra calothyrsus, I. zollingeriana=Indigofera zollingeriana

### BH of FAs

**Figure-1:**
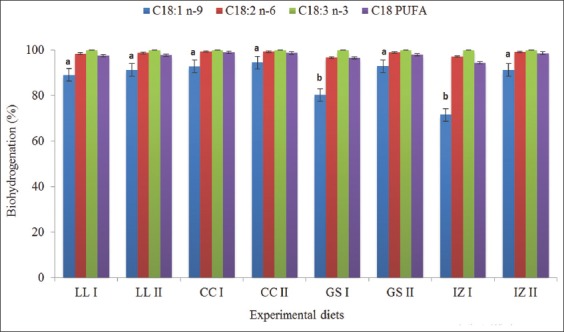
Biohydrogenation activity (%) among experimental diets. LL I=50% *Pennisetum purpureum* + 50% *Leucaena leucocephala*; LL II=75% *P. purpureum* + 25% *L. leucocephala*; GS I=50% *P. purpureum* + 50% *Gliricidia sepium*; GS II=75% *P. purpureum* + 25% *G. sepium*; IZ I=50% *P. purpureum* + 50% *I. zollingeriana*; IZ II=75% *P. purpureum* + 25% *I. zollingeriana*; CC I=50% *P. purpureum* + 50% *Calliandra calothyrsus*; CC II=75% *P. purpureum* + 25% *C. calothyrsus*. Different superscript (^a,b^) indicate significant differences (p<0.05).

**Figure-2:**
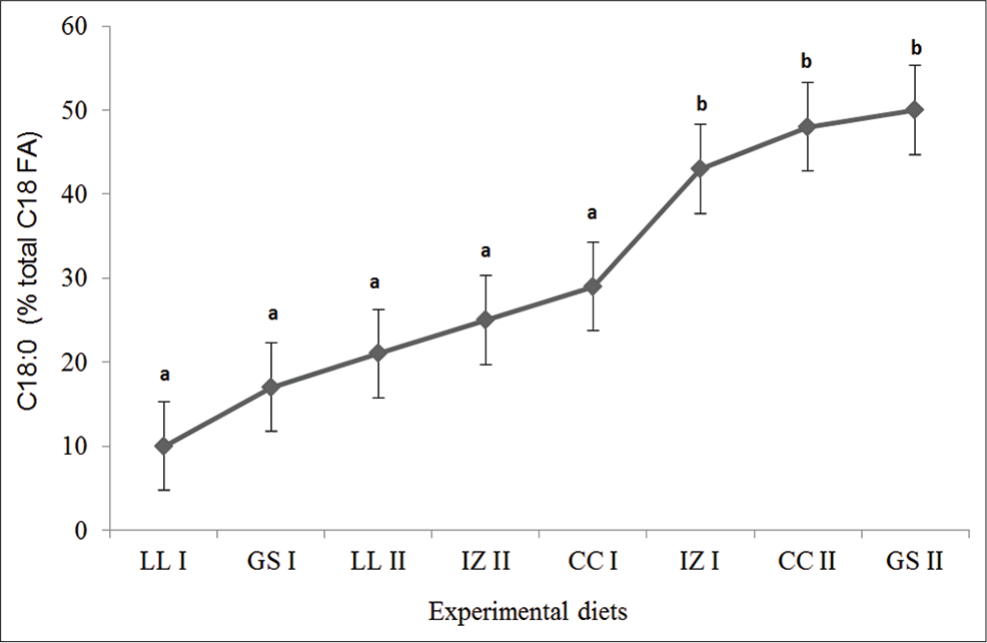
C18:0 composition of rumen fluid after 48h incubation (% total C18 FA). LL I=50% *Pennisetum purpureum* + 50% *Leucaena leucocephala*; LL II=75% *P. purpureum* + 25% *L. leucocephala*; GS I=50% *P. purpureum* + 50% *Gliricidia sepium*; GS II=75% *P. purpureum* + 25% *G. sepium*; IZ I=50% *P. purpureum* + 50% *Indigofera zollingeriana*; IZ II=75% *P. purpureum* + 25% *I. zollingeriana*; CC I=50% *P. purpureum* + 50% *Calliandra calothyrsus*; CC II=75% *P. purpureum* + 25% *C. calothyrsus*. Different superscript (^a,b^) indicate significant differences (p<0.05).

For C18:3 n-3, all experimental diets showed a BH rate of up to 100% (Figure-1). For C18:2 n-6, C18:1 n-9, and C18 PUFA, the BH rates were 97-99%, 72-95%, and 94-99%, respectively. For the appearance of C18:0, no significant difference was observed (p>0.05); a BH rate of 10-50% was observed. For the appearance of C18:0 (Figure-2), the GS II treatment ration was recorded to have the highest BH (50%), followed by CC II (48%). The lowest occurrence of C18:0(10%) was in the LL I ration, followed by the GS I (17%). No significant difference was observed (p>0.05) for the disappearance of C18:2 n-6, with the lowest loss observed in IZ I and GS I; each showed a BH rate of 97%. This trend was consistent with the BH rate of C18:1 n-9 in GS I (80%) and IZ I (72%), which showed a significant difference (p<0.05) among all groups. The appearance of *trans*-C18:2 n-6 (FA intermediate) in 48h was 0%. BH was lowest when the IZ I ration containing 50% *I. zollingeriana* was used, except when the GS I ration, containing 50% *G. sepium*, was used. The production of C18:0 was the highest when the rations containing 75% *P. purpureum*, namely, GS II and CC II, were used.

### Rumen fermentation profile

After 48h of incubation, the tropical grass-legume rations showed favorable rumen fermentation characteristics (Table-3). No significant differences, except for NH_3_ production, were observed in all treatments. The pH ranged from 6.7 to 7 and the ideal pH and total VFA values were achieved in the IZ II (7) and IZ I (6.9). NH_3_ production (mmol) was significantly different (p<0.05) among the experimental diets, and the best production was achieved using IZ I (19.7) and GS I (18.6), followed by IZ II (15.4). The *in vitro* digestibility parameters showed no significant differences (p>0.05) among treatments. The *in vitro* dry matter digestibility (IVDMD) was in the range of 57.2-67.5% and was achieved using GS II, IZ II, and LL II. The *in vitro* organic matter digestibility (IVOMD) ranged from 56.2% to 67.2%, and the best rations were GS II, IZ I, and IZ II. IVDMD and IVOMD were the lowest in CC I.

**Table-3:** Rumen fermentation profile of experimental diets after 48 h incubation.

Parameters	Experimental diets								SEM

	LL I	LL II	GS I	GS II	IZ I	IZ II	CC I	CC II	
pH	6.8	6.9	6.9	6.7	6.9	7.0	6.8	6.8	0.03
Total VFA (mmol)	118.3	111.7	112.5	99.2	120.0	125.8	114.2	117.8	2.76
NH_3_ (mmol)	12.5^a^	13.5^b^	18.6^b,c^	14.6^b^	19.7^c^	15.4^b,c^	11.2^a^	11.5^a^	1.11
IVDMD (%)	61.0	65.7	59.1	67.5	64.9	66.6	57.2	63.1	1.31
IVOMD (%)	60.7	65.4	60.3	67.3	67.2	66.9	56.2	62.1	1.44

LL I=50% *P. purpureum* + 50% *L. leucocephala*; LL II=75% *P. purpureum* + 25% *L. leucocephala*; GS I=50% *P. purpureum* + 50% *G. sepium*; GS II=75% *P. purpureum* + 25% *G. sepium*; IZ I=50% *P. purpureum* + 50% *I. zollingeriana*; IZ II=75% *P. purpureum* + 25% *I. zollingeriana*; CC I=50% *P. purpureum*+ 50% *C. calothyrsus*; CC II=75% *P. purpureum* + 25% *C. calothyrsus.* IVDMD=*In vitro* dry matter digestibility. IVOMD=*In vitro* organic matter digestibility. Different superscript (^a,b,c^) indicate significant differences (p<0.05). SEM= Standard error of the mean. *P. purpureum=Pennisetum purpureum, L. leucocephala=Leucaena leucocephala, G. sepium=Gliricidia sepium,* *C. calothyrsus=Calliandra calothyrsus, I. zollingeriana=Indigofera zollingeriana*

## Discussion

Currently, it is still a challenge to deliver beneficial FAs efficiently from ruminants to livestock products. Various approaches have been used to reduce FA metabolism and SFA intake and form an FA profile that conforms to health standards. The use of forage as the main source of PUFA for livestock and its ability to reduce BH activity through the antimicrobial activity of its phenol components makes this approach cheaper, easier to implement, and more sustainable than alternative methods. Tropical legume species are promising feed ingredients because of the ability of plants, through the *de novo* synthesis of long-chain FAs, to produce a dominant proportion of C18:3 n-3 and C18:2 n-6, followed by a small portion of C18:1 n-9 [26]. In addition, tropical legume species contain variable secondary metabolites, such as condensed tannins, hydrolyzable tannins, total phenols, polyphenol oxidase (PPO) enzymes, essential oils, and saponins, capable of modifying BH at various stages [2,9].

In this study, the differences in tropical forage species affected the FA component levels (% total FA) in their edible parts. In general, the level of C18:3 n-3 was the highest, followed by C16:0 and C18:2 n-6. *C. calothyrsus* legume species had a better FA profile than the other species. The superiority of tropical legume species over grass with respect to the FA profile was also observed. The only type of tropical grass used was *P. purpureum* and, in this grass, C16:0(22.78%) was the main FA, followed by C18:3 n-3(20.25%) and C18:2 n-6(12.66%). In this study, the FA composition was similar to that reported by Khan *et al*. [27], who measured the composition and FA content in 12 tropical forage species at various growth stages (early, medium, and late). They observed that the average FA composition (% total FA) in tropical forage is C18:3 n-3(53%), C16:0(22%), and C18:2 n-6(14%) and FA content is 8.65, 3.61, and 2.38g/kg dry matter, respectively. Jayanegara *et al*. [11] also detected C18:3 n-3 content in *L. leucocephala* and *Paspalum* grass (367 and 278g/kg FAME, respectively). However, these results contradict those of a meta-analysis study by Glasser *et al*. [28], who reported that the addition of more legume species than grass species reduces the content of C18:3 n-3 in sub-tropical pastures. This can occur because of the genetic factors of each group of plants, which have different formation performances for long-chain FAs; legume species tend to have more dominant proportions of PUFA, especially C18:3 n-3 FA, than other plant types. This is supported by a comparative study of legume species, forbs, and grass-clover mixtures that showed that legume plant species contain C18:3 n-3 FA and the highest concentrations of FA compared to the other tested plant and ration groups [29]. In addition to plant species, the composition of forage FA is influenced by factors such as: (i) Cultivars, (ii) growth stage at harvest, (iii) feed preservation treatment, and (iv) nitrogen fertilizer application [28,30].

The results of the 48h incubation on the FA metabolism of the grass-legume experimental diet showed that the BH level of each UFA was different. This may have been caused by the fact that the unsaturation level of the FA is positively correlated with rumen BH activity [31]. The C18:3 n-3 disappearance rates reached 100% and, regarding C18:2 n-6, up to 99%. This result is attributable to rumen microbial preferences for different BH processes for each component of an FA in the diet. An *in vitro* 24h study conducted by Lejonklev *et al*. [32] showed that a high rate of BH for UFA (C18:3 n-3 and C18:2 n-6) is positively correlated with the incubation period. *G. sepium* (GS I) and *I. zollingeriana* (IZ I) legumes at a 50% ratio in the experimental diet showed a significant reduction in the BH of C18:1 n-9. The results of a study by Van Ranst *et al*. [33] showed that in the 24h *in vitro* incubation of perennial ryegrass, red clover silage (50:50) provides the protective abilities of PUFAs (C18:2 n-6 and C18:3 n-3, with a BH level of 69.9% and 75.4%, respectively). This is related to the activity of the enzyme PPO, which can encapsulate lipids in plants and inhibit the first step of lipid metabolism in ruminants (lipolysis). PPO is in the sub-tropical legume species red clover (*Trifolium repens*) [34]. In tropical legumes, the total phenol content plays an important role in the modification of the metabolism of rumen FAs.

Jayanegara *et al*. [11] suggested that the total phenol content in *Persea americana* leaves is 73g/kg dry matter and these leaves are able to change the BH pattern and increase PUFA bypass and the production of *cis*-9 *trans*-11 C18:2, or conjugated linoleic acid, which has health effects. However, the total phenol content in the tropical forage species was lower than that able to modulate BH [11]. In this study, the appearance of C18:0 was not different from that in the previous studies on the use of tropical and sub-tropical plants as BH reduction agents [11,35]. *In vitro* studies have consistently shown that phenols reduce the accumulation of C18:0 and increase *trans*-11 C18:2 in the rumen digesta [9]. However, in this study, *trans*-C18:2 was not detected in the 48h period. This might have been because no intermediate FAs were available after the BH of UFA, resulting in stearic acid as the final product. This deduction is supported by Jafari *et al*. [24], who performed 24h *in vitro* experiments using goat rumen fluid and *Carica papaya* leaves as the source of polyphenols and detected the production of intermediate BH FAs (% of identified FA) such as *trans*-11 C18:1(10-14%), *cis*-*9 trans*-11 C18:2(4-6%), and *trans*-10 *cis*-12 C18:2 (<2%).

Supplementation of the ration with tropical legumes results in appropriate fermentation in the rumen. In this study, the best pH, total VFA, and NH_3_ values were achieved using the rations with *I. zollingeriana* (IZ I and IZ II). In this study, the pH range (6.9 to 7) of the rumen fluid on the basis of the *I. zollingeriana* (freshly cut) ration was at an ideal level compared to the pH ranges reported by the previous studies that used feed preservation technology in the form of Indigofera silage and pellet [36,37]. The NH_3_ production differed significantly (p<0.05) among groups and was highest in IZ I. This indicates that the inclusion of *I. zollingeriana* at a high proportion provided sufficient organic material for rumen microbes, increased the fermentability profile, and initiated an increase in the rate of the synthesis of rumen microbial proteins. Putri *et al*. [38] showed the superiority of *I. zollingeriana* over some tropical forage species (*P. purpureum*, *L. leucocephala*, and *G. sepium*); NH_3_ and total VFA contents were 36.23 mM and 162.17 mM, respectively, at pH6.85. The IVDMD and IVOMD values showed that digestibility was observed when the legume-based diet of *G. sepium* and *I. zollingeriana* was used. Suharlina *et al*. [39] reported significantly different digestibility (dry and organic matter) results for *I. zollingeriana*, *G. sepium*, and *Medicago sativa*, with the highest digestibility rates (IVDMD, 68.92%; IVOMD, >65%) achieved using *I. zollingeriana*.

## Conclusion

The various combinations of tropical legumes do not have a significant inhibitory effect on the BH of C18:2 n-6, C18:3 n-3, and C18 PUFA after *in vitro* incubation for 48h. Furthermore, an increase in the tropical legume ratio in the ration tends to suppress C18:0 formation after the fermentation simulation process. IZ I has the potential to reduce C18:1 n-9 (MUFA) disappearance and yield an ideal rumen fermentation profile.

## Authors’ Contributions

MM, MZ, FA, RS, and EMP formulated the experimental design and did experimental work at the laboratory. MM drafted the manuscript. EMP did data analysis under the guidance of MZ, FA, and RS. All the authors read and approved the final version of the manuscript.
